# *ARNT*/HIF‐1β links high‐risk 1q21 gain and microenvironmental hypoxia to drug resistance and poor prognosis in multiple myeloma

**DOI:** 10.1002/cam4.1596

**Published:** 2018-06-21

**Authors:** Chuan Wu, Ting Yang, Yingmin Liu, Yicheng Lu, Yanping Yang, Xiaobo Liu, Xuelian Liu, Long Ye, Yue Sun, Xue Wang, Qingchao Li, Peiyu Yang, Xiaoyuan Yu, Sujun Gao, Shaji Kumar, Fengyan Jin, Yun Dai, Wei Li

**Affiliations:** ^1^ Laboratory of Cancer Precision Medicine The First Hospital of Jilin University Changchun Jilin China; ^2^ Cancer Center The First Hospital of Jilin University Changchun Jilin China; ^3^ Department of Hematology Cancer Center The First Hospital of Jilin University Changchun Jilin China; ^4^ Division of Hematology Mayo Clinic College of Medicine Rochester MN USA

**Keywords:** 1q21 gain, *ARNT*/HIF‐1β, drug resistance, hypoxia, multiple myeloma

## Abstract

1q21 gain is a common cytogenetic abnormality featuring high‐risk multiple myeloma (HRMM). However, the molecular mechanism underlying the adverse prognostic effect of 1q21 gain remains largely unclear. Here, we report that *ARNT*/HIF‐1β, a 1q21 gene, is highly expressed in HRMM and induced by microenvironmental hypoxia, which confers drug resistance and correlates with inferior outcome. Analysis of the gene expression profile database revealed that *ARNT* expression was upregulated in MM and increased with disease progression or in HRMM subtypes (particularly 1q21 gain), while correlated to shorter overall survival. In a cohort of 40 MM patients, qPCR further validated that *ARNT* expression was higher in MM patients than normal donors. MM cells carrying 1q21 gain or acquired drug resistance displayed a robust increase in HIF‐1β protein level. Hypoxia induced HIF‐1β expression via a NF‐κB‐dependent process. Notably, HIF‐1β overexpression impaired bortezomib sensitivity, whereas shRNA knockdown of *ARNT* reversed hypoxia‐mediated drug resistance. Together, these findings suggest that *ARNT*/HIF‐1β might represent a novel biomarker for risk stratification and prognosis of HRMM patients, as well as a potential therapeutic target for overcoming 1q21 gain‐ or microenvironment‐mediated and acquired drug resistance in MM.

## 1 INTRODUCTION

Multiple myeloma (MM) is a malignant disease of mature plasma cells, primarily localized in bone marrow.^1^ Despite recent advances in treatment, MM remains still incurable.^2^, ^3^ Currently, the major challenges include intrinsic high‐risk disease at diagnosis^4^, ^5^ and acquired drug resistance after relapse.^6^, ^7^ In the risk stratification criteria recently updated by IMWG,^8^ MM with the unfavorable cytogenetic abnormality (CA) 1q21 gain is considered to be high risk, with poor response to standard therapies and short survival.^9^ Therefore, more precise biomarkers and therapeutic targets are urgently needed for high‐risk and relapsed/refractory MM.

As a landmark of tumor microenvironment, hypoxia is involved in virtually all aspects of cancer pathogenesis.^10^ Hypoxia triggers various cellular responses primarily via the HIF family.^11^, ^12^ HIF is a heterodimeric complex composed of one α (eg, HIF‐1α or HIF‐2α) and one β‐subunit (HIF‐1β).^11^ MM microenvironment is featured by hypoxia naturally existing in bone marrow niches where MM cells reside, which plays a critical role in MM cell survival, growth, metabolism, drug resistance, and angiogenesis, etc.^13^, ^14^, ^15^ To date, the studies of hypoxia in cancer (including MM) have focused almost exclusively on HIF‐1α, while the role of HIF‐1β remains a little known.

HIF‐1β is also known as aryl hydrocarbon receptor nuclear translocator (ARNT), a member of the basic helix‐loop‐helix/Per‐ARNT‐Sim (bHLH‐PAS) family of transcription factors. HIF‐1β predominately binds HIF‐1α or aryl hydrocarbon receptor (AhR) to form heterodimer complexes that regulate transcription of target genes involved in various physiological and pathological processes, including cancer. In the former, the role of the HIF‐1 complex consisting of HIF‐1α or ‐2α, a transactivating subunit that is inducible (eg, in response to hypoxia), and HIF1β, a regulatory subunit that is often considered as constitutively expressed and unaffected by hypoxia, has been well documented in tumor progression,^11^ tumor‐stroma interaction,^12^ metastasis,^10^ etc. In the latter, the AhR/ARNT complex binds to dioxin‐responsive elements (DREs) to induce transcription of target genes that encode drug‐metabolizing enzymes (eg, cytochrome P450 1A1) as well as proteins governing cell proliferation, differentiation, and apoptosis, which most likely facilitates carcinogenesis and tumor promotion.^16^ However, ARNT also forms a complex with AhR repressor (AhRR), which directly competes AhR/ARNT for binding to DREs and thus acts as a tumor suppressor.^16^ Moreover, ARNT might also promote survival and proliferation of tumor cells, independently of its roles in AhR and HIF signaling. For example, it has been reported that ARNT increases binding of RelB to DNA (reflecting activation of the non‐canonical NF‐κB pathway) to block the activity of RelA‐p50 dimers, indicating inhibition of the canonical NF‐κB pathway.^17^ Furthermore, unlike normal lymphocytes that express equal levels of 2 ARNT isoforms (isoforms 1 and 3), malignant lymphoid cell lines (eg, human MM and anaplastic large cell lymphoma cells) exhibit higher levels of isoform 1, which might potentiate cell proliferation by antagonizing RelB and p53‐dependent cell cycle arrest and apoptosis.^18^ Interestingly, ARNT represents a putative downstream target of the HIF‐1α/HIF‐1β complex under both hypoxic and normoxic conditions, at least in certain tumor cell lines such as Hep3B, suggesting that ARNT controls its own expression.^19^ Moreover, such an autoregulation might contribute to radioresistance.^20^ However, while the induction of ARNT by hypoxia seems like a cell type‐specific phenomenon, the regulation of ARNT remains poorly understood.

*ARNT* is located in the 1q21 region of chromosome 1, an area containing several known MM‐related genes, including *CKS1B*,* PSMD4*,* MCL1*.^21^, ^22^, ^23^ Gain of 1q21, a common adverse CA in MM, often results in amplification of these genes, which correlates to drug resistance and poor prognosis.^24^, ^25^, ^26^ However, the mechanism(s) underlying how the 1q21 genes drive disease progression and confer drug resistance remains to be defined. While information concerning *ARNT*/HIF‐1β expression in MM cells and its functional and clinical significance is lacking, we here report that *ARNT*/HIF‐1β is highly expressed in MM, particularly in high‐risk subtypes, and correlates to short survival of patients. Further, *ARNT*/HIF‐1β expression is closely associated with 1q21 gain and acquired drug resistance or can be induced by hypoxia via a NF‐κB‐dependent process. Functionally, HIF‐1β expression contributes to anti‐apoptosis and bortezomib resistance. These findings suggest *ARNT*/HIF‐1β as a novel marker for risk stratification and prognostic prediction of MM, as well as a potential target for the treatment of MM, especially the high‐risk or relapsed/refractory diseases.

## 2 MATERIALS AND METHODS

### 2.1 Cells and reagents

Human MM cells (H929, RPMI8226, U266, OPM‐2) were purchased from the ATCC and maintained as previously reported.^27^ MM cell lines acquired drug resistance were generated as described earlier.^28^ Cells were cultured in a humidified incubator at 37°C and 5% CO_2_. 1% O_2_ condition used for all hypoxic experiments was achieved in a chamber with continuous infusion of pretested gas mixture containing 95% N_2_ and 5% CO_2_. All experiments used logarithmically growing cells (3‐5 × 10^5^ cells/mL).

The NF‐κB inhibitor parthenolide^29^ and the selective IKK2 inhibitor IV^30^ were purchased from Biomol (Plymouth Meeting, PA, USA) and Calbiochem (San Diego, CA, USA), respectively. Bortezomib and lactic acid were obtained from Sigma‐Aldrich (St. Louis, MO, USA). Agents were dissolved in sterile dimethylsulfoxide (DMSO), prepared into aliquots, and stored at 20°C. Final DMSO concentrations did not exceed 0.1%.

Bone‐marrow aspirates and biopsies were obtained with informed consent from 40 patients with MM undergoing routine diagnostic procedures. CD138 cells were isolated using an immunomagnetic bead separation method, and subject to real‐time PCR analysis as described below. This study was approved by the institutional review board of the First Hospital of Jilin University.

### 2.2 Plasmids and lentiviral transfection

Human ARP‐1 MM cells, kindly provided by Dr. Wen Zhou (Central South University, Changsha, China), were transfected with construct containing human *ARNT* gene encoding the *ARNT* trasncript variant 3 in pEnter plasmid (CH816681, Vigene Biosciences, Rockville, MD, USA) and empty vector as control. Constructs encoding short hairpin RNA (shRNA) specifically targeting *ARNT*/HIF‐1β (sh*ARNT*: GAGAAGTCAGATGGTTTATTTCTC and GAGAAATAAACCATCTGACTTCTC) or a scramble sequence (shNC) were prepared using the pGreenpuro vector flagged with green fluorescent protein (GFP; Biovector Science Lab, Inc. Beijing, China). For lentiviral packing, HEK 293T cells were seeded in complete DMEM medium 1 day before transfection. Cells were then transfected with constructs encoding *ARNT*1.3 or empty vector, and shARNT or scramble shRNA along with psPAX2 and PMD2G using Lipofectamine 2000 (Invitrogen, Carlsbad, CA, USA). After 6 hours, transfection medium was replaced with fresh DMEM medium. Viral supernatants were harvested at 24 and 48 hours post‐transfection. For lentiviral infection, ARP‐1 or U266 cells were cultured with conditional medium containing viral particles for 48 hours. Transduction efficiency achieved ~70% determined by monitoring the percentage of GFP‐positive cells using flow cytometry. Subsequently, transfected cells were then selected by puromycin. Western blot analysis, qPCR, and immunofluorescent staining were performed to monitor expression of *ARNT*/HIF‐1β.

### 2.3 Analyses of cell viability and apoptosis

Cell viability was evaluated using the Cell Counting Kit‐8 (CCK8) kit (Dojindo Laboratories, Kumamoto, Japan) as per manufacturer's instruction. The percentage of apoptosis was determined by flow cytometry using Annexin V‐PE and 7AAD (BD Biosciences, San Diego, CA, USA) double staining.

### 2.4 Western blot analysis

After washed with ice‐cold PBS, whole cell lysates were prepared in RIPA lysis buffer (Cell Signalling Technology, Danvers, MA, USA) containing phenylmethylsulphonyl fluoride (PMSF) and phosphatase inhibitors (Roche, Berlin, Germany). Total protein was quantified using the Pierce BCA Protein Assay Kit (Thermo Fisher Scientific). Equal amounts of protein (30 μg) were resolved by 8% sodium dodecyl sulfate polyacrylamide gel electrophoresis (SDS‐PAGE), followed by transferring to polyvinylidene fluoride (PVDF) membranes. Membranes were blocked with PBS‐Tween‐20 containing 5% bovine serum albumin (BSA) at room temperature for 1 hour and then probed with the appropriate dilution of primary antibody overnight at 4°C, followed by incubation with a 1:5000 dilution of horseradish peroxidase‐conjugated secondary antibody (Dingguo, Beijing, China) at room temperature for 1 hour. After washing twice in PBS‐Tween‐20, the proteins were visualized using the Super Signal Chemiluminescence Kit (Thermo Fisher Scientific) by Gene Genius Bio‐Imaging System (Bio‐Rad). The following antibodies were used as primary antibodies: anti‐HIF‐1β/ARNT (rabbit), anti‐HIF‐1α (rabbit), anti‐phospho‐NF‐κB p65 (Ser 536; rabbit), and anti‐phospho‐IKKα/β (Ser 176/180; rabbit) from Cell Signalling Technology (Beverly, MA, USA); anti‐TRAF2 from BD Biosciences; anti‐CKS1B, anti‐PMSD4, anti‐Mcl‐1 from Santa Cruz Biotechnology (Dallas, TX). Where indicated, the blots were reprobed with β‐actin antibody (rabbit, Cell Signalling Technology) to ensure equal loading and transfer of proteins.

### 2.5 Qualitative real‐time PCR (qPCR)

Total RNA was extracted from cells using the Easy Pure RNA kit (Transgene Biotech, Beijing, China) as per manufacturer's instruction. One microgram per condition of total RNA was reversely transcribed to cDNA, which was then amplified with SYBR (Roche) by real‐time PCR as follows: 95°C for 10 minutes, followed by 40 cycles of 95°C for 10 seconds, and then 60°C for 30 seconds. All PCR reactions were run in triplicate, and gene expression relative to *GAPDH* was calculated using the 2^−ΔΔCT^ method.

Primers for human *ARNT* gene: forward, 5ʹ‐GGAATGGACTTGGCTCTGTAA‐3ʹ; reverse, 5ʹ‐GTCATCATCTGGGAGGAAAC‐3ʹ; the housekeeping gene *GADPH*: forward, 5ʹ‐AGAAGGCTGGGGCTCATTTG‐3ʹ; reverse, 5′‐GGATGCAGGGATGATGTTCT‐3′.

### 2.6 Statistical analysis

Values represent the means ± SD for at least 3 independent experiments performed in triplicate. The significance of differences between experimental variables was determined using the one‐way ANOVA with Tukey‐Kramer multiple comparisons test and Student's *t* test. *P *< .05 was considered significant.

## 3 RESULTS

### 3.1 *ARNT*/HIF‐1β is highly expressed in MM and correlates to high‐risk subtypes, 1q21 gain, and poor prognosis

**Figure 1:**
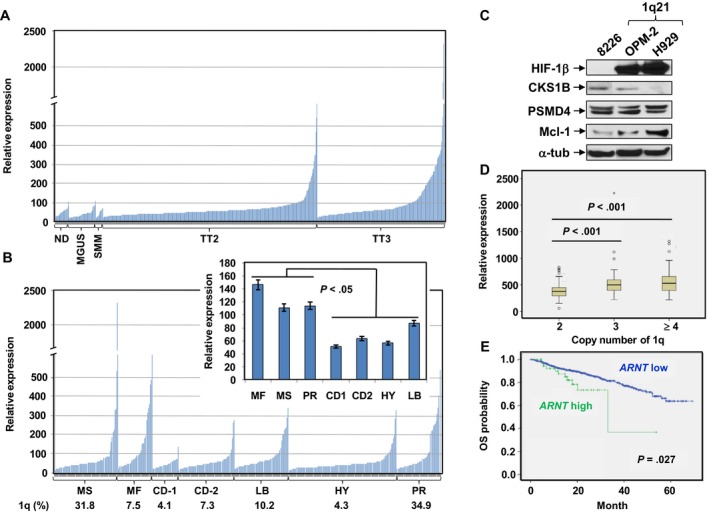
*ARNT*/HIF‐1β is highly expressed in MM, particularly in advanced disease and high‐risk subtypes, which correlates to poor prognosis. (A,B) The GEP dataset from MM patients enrolled in the Total Therapy (TT) trial was analyzed to examine the relationship between *ARNT*/HIF‐1β expression and clinical characteristics, A, expression of *ARNT* in normal donors (ND) and patients with different stages of MM, including monoclonal gammopathy of undetermined significance (MGUS), smoldering multiple myeloma (SMM), TT2 (TT + thalidomide), and TT3 (TT2 + bortezomib/Btz); and B, expression of *ARNT* in patients with high‐ and low‐risk subtypes of MM cells. According to risk stratification based on molecular classification, high‐risk MM includes MS (*MMSET*), MF (*MAF*/*MAFB*), and PR (proliferation); low‐risk MM consists of CD‐1 (*CCND1*/*CCND3*‐1), CD‐2 (*CCND1*/*CCND3*‐2), HY (hyperdiploid), LB (low bone disease). Values listed at the bottom indicate percentage of 1q21 gain in each subtype. Inset, comparison between high‐risk and low‐risk subtypes (*P *< .05). C, Western blot analysis was performed to monitor the protein levels of the key 1q21 genes, including *ARNT*/HIF‐1β, *CKS1B*, PSMD4, and *MCL1* in various MM cell lines either carrying 1q21 gain (eg, H929, OPM‐2) or not (8226). D, The GEP dataset from MM patients described in A and B was further analyzed for relation between *ARNT* expression and 1q21 copy number increase (2 copies = normal, 3 and ≥4 copies = 1q21 gain; *P *< .001 for both 3 and ≥4 copies vs 2 copies). E, Kaplan‐Meier analysis was performed to determine correlation between *ARNT* expression and overall survival (OS) in MM patients (*P* = .027 for low vs high *ARNT* expression)

To examine expression and its clinical significance of *ARNT*/HIF‐1β in MM, we first analyzed the gene expression profile (GEP) dataset of patients enrolled in the Total Therapy (TT) trial (UAMS Multiple Myeloma Database, University of Arkansas).^22^, ^24^, ^31^ As shown in Figure 1A, there was no noticeable change in *ARNT* expression between normal donors (ND) and patients with asymptomatic monoclonal gammopathy of undetermined significance (MGUS) or smoldering multiple myeloma (SMM). However, its expression was markedly increased in patients with symptomatic MM and with disease progression from TT2 (TT + thalidomide) to TT3 (TT2 + bortezomib), as well as from newly diagnosed MM to relapsed/refractory MM (Figure S1A). Moreover, high‐risk subsets (MF [*MAF*/*MAFB*], MS [*MMSET*], PR [proliferation]) exhibited significantly higher *ARNT* expression than low‐risk ones (CD‐1 [*CCND1*/*CCND3*‐1], CD‐2 [*CCND1*/*CCND3*‐2], HY [hyperdiploid], LB [low bone disease]; Figure 1B), according to the molecular criteria for risk stratification.^31^, ^32^ *ARNT* expression also correlated to adverse CAs, for example, *t*[4,14] (*FGFR3* ^+^; Figure S1B) and *t*[14,16] (*MAF* ^+^; Figure S1C) or 17p deletion (*TP53* ^+^; Figure S1D), respectively (*P *< .05 for each case).

As *ARNT* is a 1q21 gene, we then examined whether its expression would be affected by 1q21 gain, a common CA featuring a high‐risk MM subtype.^8^ Strikingly, MM cell lines carrying 1q21 gain (eg, H929 and OPM‐2) displayed a robust increase in protein level of HIF‐1β, compared to those without this CA (eg, RPMI8226, Figure 1C; U266, data not shown). However, no marked difference in proteins encoded by other 1q21 genes known to be related to poor prognosis (eg, *CKS1B*, also used as a FISH probe to detect 1q21 gain)^33^ or drug resistance (eg, PSMD4, *MCL1*)^23^, ^34^ in MM was observed. Consistently, analysis of the GEP database revealed sharply increased *ARNT* expression in MM patients carrying 1q21 gain (Figure 1D). Furthermore, Kaplan‐Meier analysis showed that patients with high *ARNT* expression had significantly shorter overall survival than those with low *ARNT* expression in all MM patients (Figure 1E), consistent with poor prognosis of MM patients carrying 1q21 gain,^24^, ^26^ as well as in the subset of patients carrying 1q21 gain (Figure S1E). These findings suggest that *ARNT*/HIF1β expression is upregulated in MM and increased with disease progression from the early precancerous stages (MGUS and SMM) to MM and with advance from NDMM to RRMM. They also raise a possibility that *ARNT*/HIF1β expression might serve as a marker for risk stratification and prediction of therapeutic response or prognosis, especially regarding high‐risk MM subtypes.

### 3.2 *ARNT*/HIF‐1β overexpression confers bortezomib resistance in MM

**Figure 2:**
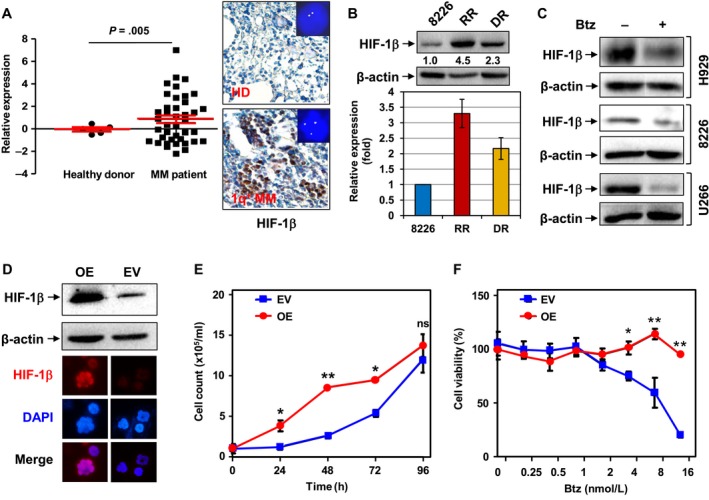
Overexpression of *ARNT*/HIF‐1β contributes to drug resistance in MM. A, *ARNT*/HIF‐1β expression was analyzed by qPCR in a cohort of patients with newly diagnosed MM (n = 40) and healthy donors (HD, n = 5). *P* = .005 for MM patients vs healthy donors (left). IHC staining for HIF‐1β as well as FISH with a probe targeting MCL1 to detect 1q21 gain were performed using bone marrow biopsies obtained undergoing routine diagnostic procedures. Representative microscopic images were shown (right). B, *ARNT*/HIF‐1β expression in drug‐naive RPMI8226 vs revlimid‐resistant (RR) and bortezomib‐resistant (DR) cells was assessed by qPCR (lower panel) and Western blot analysis (upper panel), respectively. C, MM cells (eg, H929 carrying 1q21 gain, as well as RPMI8226 and U266 without 1q21 gain) were exposed to bortezomib (Btz, 3‐5 nmol/L), for 24 h, after which Western blot analysis was performed to monitor expression of HIF‐1β. D, ARP‐1 cells, a human MM cell line, were transiently transfected with *ARNT*1.3 or empty vector (EV). Overexpression (OE) of HIF‐1β was determined by Western blot analysis (upper panel). In parallel, cells were stained for HIF‐1β by immunofluorescence (IF) and counterstained by DAPI (lower panel). Red, HIF‐1β; blue, DAPI for nucleus; bottom, merged images, indicating nuclear localization of HIF‐1β. E, ARP‐1 cells with ectopic overexpression of HIF‐1β and EV control were cultured for 4 d, and cell number was counted every 24 h (*P < .05 and **P < .01 for OE vs EV, ns = not significant). F, Alternatively, cells were exposed to a series of concentrations of Btz (0.25‐16 nmol/L) for 24 h, after which the CCK‐8 assay was performed to determine cell viability (*P < .05 and **P < .01 for OE vs EV). For E and F, values represent mean ± SD for at least 3 independent experiments performed in triplicate, respectively

Unlike HIF‐1α that has been extensively investigated,^35^ it is little known about the function of its regulatory partner HIF‐1β in cancer, including MM. To explore the functional role of HIF‐1β in MM, we first validated *ARNT* expression in a cohort of 40 MM patients. Consistent with the analysis of the GEP database described above, *ARNT* was significantly upregulated in CD138^+^ cells isolated from bone marrow samples of MM patients (Figure 2A, left). Moreover, *ARNT* upregulation was observed in 66.7% (10/15) of RRMM patients, higher than 48% (12/25) for NDMM patients, suggesting a possible role of *ARNT* expression in drug resistance after relapsed on current frontline treatment. In addition, IHC revealed that the protein level of HIF‐1β was markedly increased in bone marrow biopsies of MM patients, particularly those carrying 1q21 gain, compared to healthy donors (Figure 2A, right; inset, FISH for detection of 1q21 gain; the representative areas shown are indicated by square in Figure S2A). *ARNT*/HIF‐1β expression was then compared between RPMI8226 cells and their counterparts acquired drug resistance to either bortezomib (DR) or lenalidomide (RR). Notably, both mRNA and protein levels of *ARNT*/HIF‐1β were markedly increased in both DR and RR cells, compared to drug‐naive parental cells (Figure 2B). Moreover, almost identical results were obtained in another MM cell line (PS‐R) acquired bortezomib resistance, compared to parental U266 cells (Figure S2B). Significantly, while exposure to bortezomib clearly downregulated HIF‐1β expression in various MM cell lines (eg, H929, RPMI8226, U266), ectopic *ARNT* overexpression, manifested by increased HIF‐1β protein level (Figure 2C, upper) and increased fluorescent signal in the nucleus (Figure 2C, lower; Figure S2C), promoted cell growth (Figure 2D) and reduced bortezomib lethality (Figure 2E). These findings indicate that HIF‐1β is markedly upregulated in MM cells, which functionally confers bortezomib resistance. They also suggest that increased HIF‐1β expression might be associated to acquired drug resistance toward bortezomib and lenalidomide, which might explain, at least in part, why RRMM patients often respond poorly to these agents.

### 3.3 Hypoxia induces *ARNT*/HIF‐1β expression, in association with NF‐κB activation

**Figure 3:**
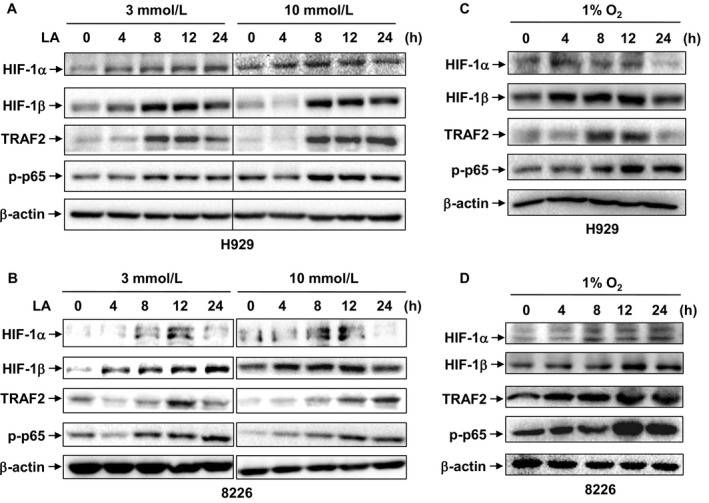
Hypoxia induces *ARNT*/HIF‐1β expression, accompanied by NF‐κB activation. (A,B) H929 cells carrying 1q21 gain (A) and RPMI8226 cells without 1q21 gain (B) were exposed to lactic acid (LA, 3 mmol/L or 10 mmol/L) for the indicated intervals (4‐24 h). (C,D) H929 (C) and RPMI8226 cells (D) were cultured under hypoxia (1% O_2_) for 4‐24 h. After treatment, Western blot analysis was performed to monitor expression of HIF‐1α, HIF‐1β, TRAF2, as well as phosphorylation of p65 (S536). Representative results of 3 independent experiments were shown

HIF‐1, a complex composed of HIF‐1α and HIF‐1β, and NF‐κB (p65/p50 as the most abundant form) represent 2 major transcription factors in response to hypoxia.^36^ In this context, we then examined whether hypoxia activates these 2 signaling pathways in MM cells. As shown in Figure 3, either exposure to the hypoxic mimetic lactic acid (3 mmol/L or 10 mmol/L) or hypoxia (1% O_2_) induced a time‐dependent expression of HIF‐1α and HIF‐1β, accompanied by NF‐κB activation (reflected by upregulation of TRAF2, a key component of the NF‐κB pathway,^37^ and S536 phosphorylation of p65, catalyzed by activated IKK‐β 30) in H929 (Figure 3A,B), RPMI8226 (Figure 3C,D), and U266 cells (Figure S3A). However, treatment with CoCl_2_, a chemical approach widely used to mimic hypoxia, failed to induce either HIF‐1β expression or NF‐κB activation in MM cells, while resulted in HIF‐1α accumulation (Figure S3B), presumably due to blockade of its degradation.^13^, ^38^ These results indicate that hypoxia (eg, low O_2_ concentration or the chemical mimetic lactic acid, but not CoCl_2_) is able to induce *ARNT*/HIF‐1β expression and NF‐κB activation in MM cells, suggesting a potential role of HIF‐1β and its relationship with NF‐κB in hypoxic MM microenvironment.

### 3.4 Hypoxia contributes to microenvironment‐mediated bortezomib resistance

**Figure 4:**
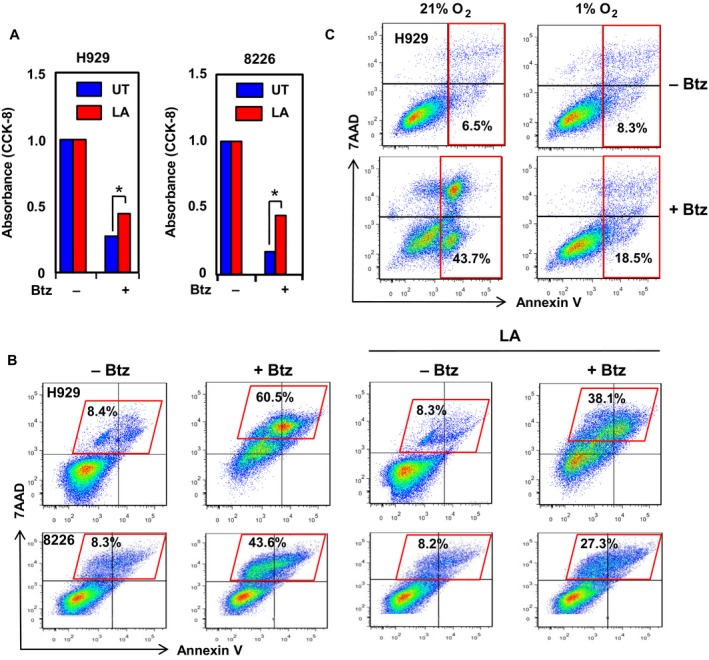
Hypoxia reduces bortezomib sensitivity in MM cells. A, H929 and RPMI8226 cells were preincubated with 3 mmol/ml lactic acid (LA) for 8 h, followed by exposure to 3 nmol/L (H929) or 5 nmol/L (8226) bortezomib (Btz) for additional 24 h, after which the CCK‐8 assay was performed to determine cell viability (*P < .05 for LA vs UT in each cell line). UT, untreated control. Values represent mean ± SD for at least 3 independent experiments performed in triplicate, respectively. B, Alternatively, flow cytometric analysis was performed to determine percentage of cell death (in parallelogram, including Annexin V/7AAD double positive and 7AAD single positive cells) after stained with Annexin V‐FITC and 7AAD. C, H929 cells were precultured under hypoxia (1% O_2_) for 4 h, followed by treatment with 3 nmol/L Btz for additional 16 h, after which the percentage of apoptotic cells (in rectangle, including Annexin V single positive and Annexin V/7AAD double positive) was determined by flow cytometry. For B and C, representative data of at least 3 independent experiments were shown

Hypoxia naturally existed in bone marrow niche where MM cells reside, providing microenvironment for MM cells to survive and grow, as well as to escape the lethal action of anti‐MM agents. To examine whether hypoxia is related with drug resistance, MM cells were preincubated with lactic acid for 8 hours when HIF‐1β expression was markedly induced as shown above (Figure 3A,C), followed by exposure to bortezomib for additional 24 hours. The CCK‐8 assay revealed that preincubation with lactic acid partially, but significantly, increased cell viability after treated with bortezomib, in H929 and RPMI8226 cells (Figure 4A; *P *< .05 for each case). Moreover, flow cytometric analysis demonstrated that pretreatment with lactic acid significantly prevented bortezomib‐induced apoptosis in H929 and RPMI8226 cells (representative results shown in Figure 4B, *P *< .01 for at least 3 independent experiments performed in triplicate). Similarly, 8 hours preincubation under hypoxia also markedly reduced apoptosis induced by bortezomib in H929 cells (representative results shown in Figure 4C, *P *< .01 for at least 3 independent experiments performed in triplicate), and to a lesser extent in RPMI8226 cells (*P *< .05, Figure S3C). Taken together, these findings indicate that hypoxia is functionally involved in microenvironment‐mediated bortezomib resistance, likely in association with activation of the HIF and NF‐κB pathways.

### 3.5 *ARNT*/HIF‐1β expression depends on NF‐κB activation in MM cells

**Figure 5:**
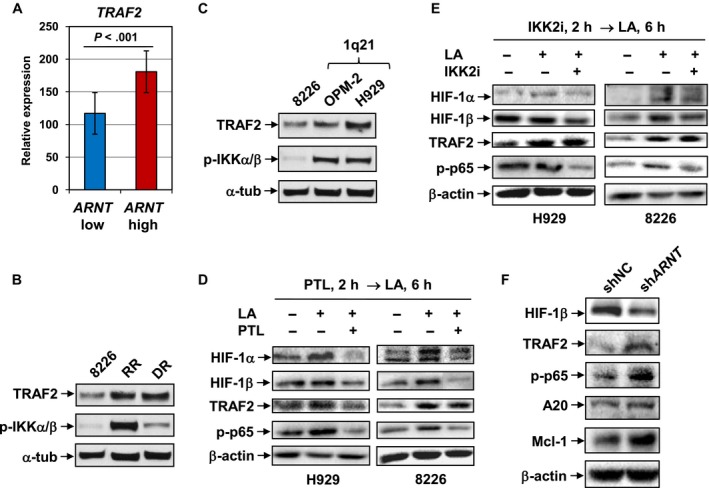
*ARNT*/HIF‐1β expression is dependent on NF‐κB activation. A, The GEP dataset from MM patients described in Figure 1E was analyzed for relationship between *ARNT* and *TRAF2* expression in MM patients (*P *< .001 for low vs high *ARNT* groups). (B,C) Expression of TRAF2 and phosphorylation of IKKα/β (S176/180) were examined by Western blot analysis in drug‐naive and drug‐resistant cell lines (B) as described in Figure 2B, as well as various MM cell lines with or without 1q21 gain (C) as described in Figure 1C. (D,E) H929 (left) and RPMI8226 cells (right) were pretreated with either 10 μmol/L parthenolide (PTL; D) or 10 μmol/L IKK2 inhibitor (IKK2i; E) for 2 h, followed by exposure to 3 mmol/L lactic acid (LA) for additional 6 h. After treatment, Western blot analysis was performed to monitor the protein levels of HIF‐1α, HIF‐1β, and TRAF2, as well as phosphorylation of p65 (S536). F, U266 cells were stably transfected with pGreenpuro‐shRNA constructs encoding shRNA targeted *ARNT* (sh*ARNT*) or scramble sequence as negative control (shNC), in which expression of HIF‐1β, TRAF2, TNFAIP3/A20, and Mcl‐1, as well as phosphorylation of p65 (S536) was examined by Western blot analysis. For B‐F, representative results of at least 3 independent experiments were shown

Cross‐talk between HIF (HIF‐1α in particular) and NF‐κB is well‐established in certain physiological circumstances such as inflammation and immune response.^35^, ^36^, ^39^ As hypoxia‐induced expression of HIF‐1α and HIF‐1β was accompanied by NF‐κB activation (Figure 3), a possibility then arose that these 2 pathways might communicate to each other in MM cells, particularly under hypoxia within bone marrow microenvironment. To this end, we next examined the functional relationship between *ARNT*/HIF1β expression and NF‐κB activation in MM cells. As shown in Figure 5A, analysis of the GEP database revealed a significant correlation between *ARNT* and *TRAF2* in primary MM samples. *ARNT*/HIF‐1β expression was also associated with NF‐κB activation, reflected by TRAF2 upregulation and IKKα/β phosphorylation, in MM cells either acquired drug resistance (eg, RR and DR cells; Figure 5B; parallel HIF‐1β expression shown in Figure 2B) or carrying 1q21 gain (eg, OPM‐2 and H929 cells; Figure 5C; parallel HIF‐1β expression shown in Figure 1C).

To test the functional role of NF‐κB in regulation of HIF‐1β expression, a pan NK‐κB inhibitor (parthenolide, PTL)^29^ and a selective IKK‐β inhibitor (IKK2i)^30^ were employed to block activation of the NF‐κB pathway. As shown in Figure 5D,E, pretreatment with either PTL (Figure 5D) or IKK2i (Figure 5E) for 2 hours prevented p65 phosphorylation in H929 and RPMI8226 cells exposed to lactic acid, while TRAF2 was downregulated by PTL, but not by IKK2i that specifically inhibits IKK‐β kinase activity.^30^ Nevertheless, lactic acid‐induced expression of HIF‐1α and HIF‐1β was markedly blocked by pretreatment with PTL and to a lesser extent IKK2i. However, treatment with PTL or IKK2i (Figure S3D) after 6 hours preincubation with lactic acid failed to diminish lactic acid‐induced HIF‐1β expression, although NF‐κB activation was inhibited, presumably because *ARNT*/HIF‐1β has already been induced. Conversely, HIF‐1β knockdown by *ARNT* shRNA did not attenuate, rather increased basal NF‐κB activity (eg, *TRAF2* expression and p65 phosphorylation; Figure 5F), as well as upregulated its target genes (eg, *TNFAIP3*/A20, *MCL1*, etc.), probably representing a compensatory response to HIF‐1β downregulation. Together, these results indicate that HIF‐1β expression is closely associated with NF‐κB activation in MM cells, especially those carrying 1q21 gain or acquired drug resistance. They also suggest that hypoxia implicating bone marrow microenvironment induced HIF‐1β expression via a NF‐κB‐dependent process, supporting *ARNT*/HIF‐1β as a downstream target of NF‐κB in MM cells.

### 3.6 *ARNT*/HIF‐1β functionally contributes to bortezomib resistance

**Figure 6:**
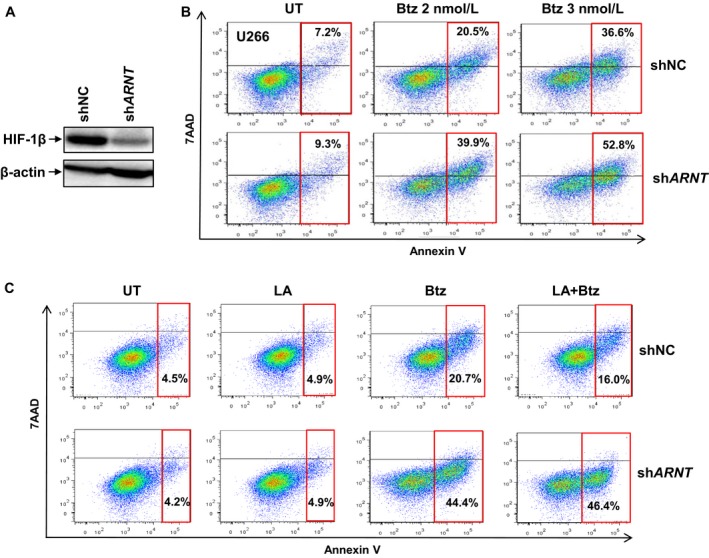
Knockdown of *ARNT*/HIF‐1β sensitizes MM cells to bortezomib and abrogates the cytoprotective effect of hypoxia. A, U266 cells were stably transfected with pGreenpuro‐shRNA constructs encoding shRNA targeted *ARNT* (sh*ARNT*) or scramble sequence as negative control (shNC), in which expression of HIF‐1β was examined by Western blot analysis to validate knockdown of *ARNT* expression. (B,C) Cells were then exposed to 2‐3 nmol/L bortezomib (Btz) for 24 h (B) or preincubated with lactic acid (LA) for 8 h, followed by treatment with Btz for additional 24 h (C). After treatment, percentage of apoptotic cells (in rectangle, including Annexin V single positive and Annexin V/7AAD double positive) was determined by flow cytometry. For B and C, representative data of at least 3 independent experiments were shown

Last, we examined the functional role of *ARNT*/HIF‐1β in regulation of bortezomib sensitivity and hypoxia‐mediated drug resistance. Western blot analysis was first performed to confirm HIF‐1β downregulation by sh*ARNT* by comparing to control shRNA targeting scramble sequence (Figure 6A). Notably, HIF‐1β knockdown significantly sensitized MM cells to bortezomib (representative results shown in Figure 6B for at least 3 experiments performed in triplicate; *P *< .01 or *P *< .05 for 2 and 3 nmol/L bortezomib, respectively). Moreover, while 8 hour preincubation with lactic acid moderately but clearly prevented apoptosis induced by bortezomib, this cytoprotective effect was completely abrogated by shRNA knockdown of HIF‐1β (representative results shown in Figure 6C for at least 3 experiments performed in triplicate; *P *< .001 for sh*ARNT* vs shNC cells after treated with lactic acid + bortezomib, while *P* > .05 for bortezomib vs lactic acid + bortezomib in sh*ARNT* cells). Taken together, these results suggest that as *ARNT*/HIF‐1β is overexpressed in high‐risk MM subtypes and RRMM acquired drug resistance, or induced by hypoxia in bone marrow microenvironment, it functionally contributes to drug resistance, thereby most likely accounting for poor response of patients with high‐risk MM or RRMM to the frontline agents such as bortezomib. They also raise a possibility that targeting *ARNT*/HIF‐1β might be able to overcome drug resistance in high‐risk MM and RRMM or conferred by microenvironment, in order to improve clinical outcome of those hard‐to‐treat MM patients who currently display poor prognosis due to lack of effective therapy.

## 4 DISCUSSION

Multiple myeloma is characterized by the heterogeneity of cytogenetic abnormalities (CAs), including copy number variation, amplification/deletions of genomic fragments, and even loss of chromosome arms or whole chromosomes.^1^ Such heterogeneity accounts for the complexity of this diseases in pathogenesis and progression, as well as diagnosis and treatment. MM patients carrying certain types of CAs fail to or poorly respond to the current frontline therapy, leading to dismal outcomes. In the criteria recently recommended by IMWG, the high‐risk CAs include *t*(4;14), *t*(14;16), *t*(14;20), del(17/17p), and gain(1q).^8^ Among them, gain(1q), gain of 1q21 region, is common (~40% at diagnosis and over 70% at relapse) and associated with poor prognosis of MM patients even in the era of novel agents.^24^, ^26^ 1q21 gain results in amplification or overexpression of several 1q21 genes (eg, *CKS1B*,* MCL1*,* PSMD4*,* ANP32E*) and is thus considered as a diver CA in MM.^40^ However, although some of them (eg, *CKS1B*,* MCL1*) have been used for FISH probes to detect 1q21 gain,^33^ little is known about how these or other 1q21 genes drive disease progression or confer drug resistance.^41^ In this study, we identified *ARNT*/HIF‐1β, another 1q21 gene, as a prognostic marker as well as therapeutic target for high‐risk MM (eg, 1q21 gain) and RRMM acquired drug resistance, particularly regarding hypoxic bone marrow microenvironment.

To date, outcome of MM patients carrying 1q21 gain remain poor, primarily due to failure to overcome drug resistance stemmed from this adverse CA.^42^, ^43^ To serve as a prognostic marker for this high‐risk subtype of MM, at least 3 criteria should be met, including that (1) it is often upregulated in MM and better increased with disease progression; (2) it must be amplified and/or overexpressed due to 1q21 gain; and (3) its expression must significantly correlate with poor prognosis of patients.^44^ Moreover, it would be more valuable if such a gene also functionally contributes to disease progression and/or drug resistance and thereby serves as a therapeutic target as well. *CKS1B* has been used the most widely to detect 1q21 gain and when it is ectopically overexpressed, promotes proliferation and prevents apoptosis of MM cells via p27‐dependent and independent processes.^45^ However, *CKS1B* amplification or overexpression by itself might not correlate to short survival of MM patients.^46^ It is also noteworthy that *CKS1B* expression has been determined exclusively at mRNA level so far,^47^ which does not necessarily reflect its protein level and function. In fact, no increase in protein level of *CKS1B*, neither other known MM‐related 1q21 genes (eg, *PSMD4*,* MCL1*) was observed in human MM cell lines carrying 1q21 gain. In contrast, it was noted that the protein level of *ARNT*/HIF‐1β was extremely high in these cells. Similar to *CKS1B*, ectopic expression of *ARNT*/HIF1β also increased MM cell growth and led to bortezomib resistance, while knockdown of its endogenous level significantly sensitized MM cells to this agent. Therefore, these findings argue that the 1q21 gene *ARNT* meets all criteria for a biomarker of high‐risk MM carrying 1q21 gain. They also suggest that *ARNT*/HIF‐1β might serve as a potential target to overcome drug resistance stemmed from 1q21 gain. However, like *CKS1B*,* ARNT* likely not represent a sole marker for diagnosis and risk stratification of MM patients carrying 1q21 gain, due to heterogeneity of its expression in this subset of MM. To this end, GEP targeting a panel of multiple genes (1q21 genes in particular) might be required for more precise risk stratification.

MM microenvironment is featured by hypoxia that naturally exists in bone marrow niche where MM cells reside.^48^ Moreover, hypoxia is known to play an important role in MM cell survival and growth, disease progression, and drug resistance.^49^ Hypoxic responses are primarily mediated by the HIF family including HIF‐1α, HIF‐2α, HIF‐3α, and HIF‐1β. To be an active transcription factor, HIF‐1α must form a complex with HIF‐1β. In normoxia, HIF‐1α rapidly turns over via ubiquitin‐proteasome system (UPS), while under hypoxia, it translocates to the nucleus where it forms a heterodimer with HIF‐1β to trigger transcription of target genes.^11^ Therefore, HIF‐1α is inducible and unstable, while HIF‐1β is often considered to be constitutively expressed. Of note, we observed that hypoxia (or lactic acid, a cellular metabolic product under hypoxia) induced marked expression of *ARNT*/HIF‐1β in MM cells. Moreover, this event reduced bortezomib sensitivity, which was largely reversed by HIF‐1β knockdown, thereby supporting the functional role of HIF‐1β in hypoxic MM microenvironment. Interestingly, unlike HIF‐1α that accumulates after exposed to bortezomib due to inhibition of its degradation via UPS, HIF‐1β was sharply downregulated after treatment with bortezomib. In this context, cobalt (CoCl_2_), an approach widely used to mimic hypoxia, stabilizes HIF‐1α through inhibition of its hydroxylation mediated by PHDs and following degradation via UPS.^50^ However, cobalt failed to induce HIF‐1β expression in MM cells, suggesting that this HIF‐1α inducer might not be an optimal approach to mimic hypoxic microenvironment in MM. Indeed, we also found that cobalt, unlike hypoxia or lactic acid, did not protect MM cells from apoptosis induced by bortezomib, either (data not shown). In contrast, it has been observed that expression of *ARNT* could be un‐regulated at both mRNA and protein levels by hypoxia or hypoxic mimetics (eg, CoCl_2_ and dimethyloxalylglycine/DMOG) in a cell line‐specific manner.^51^ However, this event is largely diminished by siRNA knockdown of *HIF‐1A*,^51^ arguing that it might be secondary to HIF activation due to HIF‐1α accumulation, consistent with the notion that *ARNT* is a putative target gene of HIF signaling.^19^

NF‐κB is another major transcriptional factor in response to hypoxia.^36^ Whereas cross‐talk between HIF‐1α and NF‐κB has been well documented,^35^, ^36^, ^39^ it is little known about relationship between HF‐κB and HIF‐1β. In endothelial cells, we have recently demonstrated that *ARNT*/HIF‐1β expression is NF‐κB dependent, suggesting HIF‐1β as a downstream target of NF‐κB.^52^ In the present study, we also observed (1) a close association of *ARNT*/HIF‐1β expression with NF‐κB activation in MM cells carrying 1q21 gain, acquired drug resistance (eg, toward bortezomib or lenalidomide), or exposed to hypoxia (or lactic acid); (2) correlation in gene expression between *ARNT*/HIF‐1β and TRAF2, a key component of the NF‐κB pathway, in primary MM samples; and more directly (3) attenuation of hypoxia‐induced *ARNT*/HIF‐1β expression by NF‐κB inhibition, but failure to affect NF‐κB activation by *ARNT*/HIF‐1β knockdown. Thus, although HIF‐1α accumulation could compensate lethal action of bortezomib, this agent might impair transcriptional activity of the HIF complex by downregulation of its regulatory subunit *ARNT*/HIF1β through NF‐κB inhibition. This might provide a new insight into mechanism of action or drug resistance for bortezomib, and probably other PIs (eg, carfilzomib and ixazomib) as well.

In summary, we identify for the first time, to the best of our knowledge, *ARNT*/HIF‐1β as a potential biomarker for prediction of therapeutic response and prognosis of MM patients, especially those carrying high‐risk 1q21 gain. Hypoxia or lactic acid (but not cobalt) induces *ARNT*/HIF‐1β expression via a NF‐κB‐dependent process, implicating in MM microenvironment. HIF‐1β might also represent a potential target for treatment of MM carrying adverse CAs (eg, 1q21 gain) or acquired drug resistance, as well as for overcoming microenvironment‐mediated drug resistance. Together, *ARNT*/HIF‐1β warrants further investigation in risk stratification and treatment of MM, particularly hard‐to‐treat diseases.

## CONFLICT OF INTEREST

The authors declare no conflict of interest.

## References

[1] Kumar SK , Rajkumar V , Kyle RA , et al. Multiple myeloma. Nat Rev Dis Primers. 2017;3:17046. 28726797 10.1038/nrdp.2017.46

[2] Orlowski RZ , Lonial S . Integration of novel agents into the care of Patients with multiple myeloma. Clin Cancer Res. 2016;22:5443‐5452. 28151712 10.1158/1078-0432.CCR-16-0861 PMC5705234

[3] Anderson KC . Progress and paradigms in multiple myeloma. Clin Cancer Res. 2016;22:5419‐5427. 28151709 10.1158/1078-0432.CCR-16-0625 PMC5300651

[4] Usmani SZ , Rodriguez‐Otero P , Bhutani M , Mateos MV , Miguel JS . Defining and treating high‐risk multiple myeloma. Leukemia. 2015;29:2119‐2125. 26265183 10.1038/leu.2015.209

[5] Dispenzieri A . Myeloma: management of the newly diagnosed high‐risk patient. Hematology Am Soc Hematol Educ Program. 2016;2016:485‐894. 27913520 10.1182/asheducation-2016.1.485 PMC6142458

[6] Nooka AK , Kastritis E , Dimopoulos MA , Lonial S . Treatment options for relapsed and refractory multiple myeloma. Blood. 2015;125:3085‐3099. 25838342 10.1182/blood-2014-11-568923

[7] Harousseau JL , Attal M . How I treat first relapse of myeloma. Blood. 2017;130:963‐973. 28679737 10.1182/blood-2017-03-726703

[8] Sonneveld P , Avet‐Loiseau H , Lonial S , et al. Treatment of multiple myeloma with high‐risk cytogenetics: a consensus of the International Myeloma Working Group. Blood. 2016;127:2955‐2962. 27002115 10.1182/blood-2016-01-631200 PMC4920674

[9] Rajkumar SV . Multiple myeloma: 2016 update on diagnosis, risk‐stratification, and management. Am J Hematol. 2016;91:719‐734. 27291302 10.1002/ajh.24402 PMC5291298

[10] Rankin EB , Giaccia AJ . Hypoxic control of metastasis. Science. 2016;352:175‐180. 27124451 10.1126/science.aaf4405 PMC4898055

[11] Schito L , Semenza GL . Hypoxia‐inducible factors: master regulators of cancer progression. Trends Cancer. 2016;2:758‐770. 28741521 10.1016/j.trecan.2016.10.016

[12] LaGory EL , Giaccia AJ . The ever‐expanding role of HIF in tumour and stromal biology. Nat Cell Biol. 2016;18:356‐365. 27027486 10.1038/ncb3330 PMC4898054

[13] Colla S , Storti P , Donofrio G , et al. Low bone marrow oxygen tension and hypoxia‐inducible factor‐1alpha overexpression characterize patients with multiple myeloma: role on the transcriptional and proangiogenic profiles of CD138(+) cells. Leukemia. 2010;24:1967‐1970. 20811474 10.1038/leu.2010.193

[14] Martin SK , Diamond P , Gronthos S , Peet DJ , Zannettino AC . The emerging role of hypoxia, HIF‐1 and HIF‐2 in multiple myeloma. Leukemia. 2011;25:1533‐1542. 21637285 10.1038/leu.2011.122

[15] Ria R , Catacchio I , Berardi S , et al. HIF‐1alpha of bone marrow endothelial cells implies relapse and drug resistance in patients with multiple myeloma and may act as a therapeutic target. Clin Cancer Res. 2014;20:847‐858. 24297864 10.1158/1078-0432.CCR-13-1950

[16] Vogela CFA , Haarmann‐Stemmannb T . The aryl hydrocarbon receptor repressor ‐ More than a simple feedback inhibitor of AhR signaling: clues for its role in inflammation and cancer. Curr Opin Toxicol. 2017;2:109‐119. 28971163 10.1016/j.cotox.2017.02.004 PMC5621755

[17] Wright CW , Duckett CS . The aryl hydrocarbon receptor nuclear translocator alters CD30‐mediated NF‐κB‐dependent transcription. Science. 2009;323:251‐255. 19131627 10.1126/science.1162818 PMC2682336

[18] Gardella KA , Muro I , Fang G , Sarkar K , Mendez O , Wright CW . Aryl hydrocarbon receptor nuclear translocator (ARNT) isoforms control lymphoid cancer cell proliferation through differentially regulating tumor suppressor p53 activity. Oncotarget. 2016;7:10710‐10722. 26909609 10.18632/oncotarget.7539 PMC4905433

[19] Mandl M , Depping R . ARNT is a potential direct HIF‐1 target gene in human Hep3B hepatocellular carcinoma cells. Cancer Cell Int. 2017;17:77. 28855849 10.1186/s12935-017-0446-2 PMC5571568

[20] Mandl M , Lieberum M‐K , Dunst J , Depping R . The expression level of the transcription factor Aryl hydrocarbon receptor nuclear translocator (ARNT) determines cellular survival after radiation treatment. Radiat Oncol. 2015;10:229. 26572229 10.1186/s13014-015-0539-9 PMC4647475

[21] Chang H , Qi X , Trieu Y , et al. Multiple myeloma patients with CKS1B gene amplification have a shorter progression‐free survival post‐autologous stem cell transplantation. Br J Haematol. 2006;135:486‐491. 16995883 10.1111/j.1365-2141.2006.06325.x

[22] Shaughnessy JD Jr , Zhan F , Burington BE , et al. A validated gene expression model of high‐risk multiple myeloma is defined by deregulated expression of genes mapping to chromosome 1. Blood. 2007;109:2276‐2284. 17105813 10.1182/blood-2006-07-038430

[23] Shaughnessy JD Jr , Qu P , Usmani S , et al. Pharmacogenomics of bortezomib test‐dosing identifies hyperexpression of proteasome genes, especially PSMD4, as novel high‐risk feature in myeloma treated with Total Therapy 3. Blood. 2011;118:3512‐3524. 21628408 10.1182/blood-2010-12-328252 PMC3186329

[24] Hanamura I , Stewart JP , Huang Y , et al. Frequent gain of chromosome band 1q21 in plasma‐cell dyscrasias detected by fluorescence in situ hybridization: incidence increases from MGUS to relapsed myeloma and is related to prognosis and disease progression following tandem stem‐cell transplantation. Blood. 2006;108:1724‐1732. 16705089 10.1182/blood-2006-03-009910 PMC1895503

[25] Wang W , Zhang Y , Chen R , et al. Chromosomal instability and acquired drug resistance in multiple myeloma. Oncotarget. 2017;8:78234‐78244. 29100463 10.18632/oncotarget.20829 PMC5652852

[26] Avet‐Loiseau H , Attal M , Campion L , et al. Long‐term analysis of the IFM 99 trials for myeloma: cytogenetic abnormalities [t(4;14), del(17p), 1q gains] play a major role in defining long‐term survival. J Clin Oncol. 2012;30:1949‐1952. 22547600 10.1200/JCO.2011.36.5726

[27] Dai Y , Landowski TH , Rosen ST , Dent P , Grant S . Combined treatment with the checkpoint abrogator UCN‐01 and MEK1/2 inhibitors potently induces apoptosis in drug‐sensitive and ‐resistant myeloma cells through an IL‐6‐independent mechanism. Blood. 2002;100:3333‐3343. 12384435 10.1182/blood-2002-03-0940

[28] Chen S , Zhang Y , Zhou L , et al. A Bim‐targeting strategy overcomes adaptive bortezomib resistance in myeloma through a novel link between autophagy and apoptosis. Blood. 2014;124:2687‐2697. 25208888 10.1182/blood-2014-03-564534 PMC4208284

[29] Dai Y , Guzman ML , Chen S , et al. The NF (Nuclear factor)‐ κB inhibitor parthenolide interacts with histone deacetylase inhibitors to induce MKK7/JNK1‐dependent apoptosis in human acute myeloid leukaemia cells. Br J Haematol. 2010;151:70‐83. 20701602 10.1111/j.1365-2141.2010.08319.x PMC2950247

[30] Dai Y , Chen S , Wang L , et al. Disruption of IκB kinase (IKK)‐mediated RelA serine 536 phosphorylation sensitizes human multiple myeloma cells to histone deacetylase (HDAC) inhibitors. J Biol Chem. 2011;286:34036‐34050. 21816815 10.1074/jbc.M111.284216 PMC3190767

[31] Zhan F , Huang Y , Colla S , et al. The molecular classification of multiple myeloma. Blood. 2006;108:2020‐2028. 16728703 10.1182/blood-2005-11-013458 PMC1895543

[32] Szalat R , Avet‐Loiseau H , Munshi NC . Gene expression profiles in myeloma: ready for the real world? Clin Cancer Res. 2016;22:5434‐5442. 28151711 10.1158/1078-0432.CCR-16-0867 PMC5546147

[33] Chang H , Yeung J , Xu W , Ning Y , Patterson B . Significant increase of CKS1B amplification from monoclonal gammopathy of undetermined significance to multiple myeloma and plasma cell leukaemia as demonstrated by interphase fluorescence in situ hybridisation. Br J Haematol. 2006;134:613‐615. 16889615 10.1111/j.1365-2141.2006.06237.x

[34] Zhang Y , Zhou L , Leng Y , Dai Y , Orlowski RZ , Grant S . Positive transcription elongation factor b (P‐TEFb) is a therapeutic target in human multiple myeloma. Oncotarget. 2017;8:59476‐59491. 28938651 10.18632/oncotarget.19761 PMC5601747

[35] Triner D , Shah YM . Hypoxia‐inducible factors: a central link between inflammation and cancer. J Clin Invest. 2016;126:3689‐3698. 27525434 10.1172/JCI84430 PMC5096825

[36] D'Ignazio L , Batie M , Rocha S . Hypoxia and inflammation in cancer, focus on HIF and NF‐κB. Biomedicines. 2017;5:E21. 28536364 10.3390/biomedicines5020021 PMC5489807

[37] Demchenko YN , Glebov OK , Zingone A , Keats JJ , Bergsagel PL , Kuehl WM . Classical and/or alternative NF‐κB pathway activation in multiple myeloma. Blood. 2010;115:3541‐3552. 20053756 10.1182/blood-2009-09-243535 PMC2867265

[38] Jaakkola P , Mole DR , Tian YM , et al. Targeting of HIF‐alpha to the von Hippel‐Lindau ubiquitylation complex by O_2_‐regulated prolyl hydroxylation. Science. 2001;292:468‐472. 11292861 10.1126/science.1059796

[39] Rius J , Guma M , Schachtrup C , et al. NF‐κB links innate immunity to the hypoxic response through transcriptional regulation of HIF‐1alpha. Nature. 2008;453:807‐811. 18432192 10.1038/nature06905 PMC2669289

[40] Sonneveld P . Gain of 1q21 in multiple myeloma: from bad to worse? Blood. 2006;108:1426‐1427.

[41] Marchesini M , Ogoti Y , Fiorini E , et al. ILF2 is a regulator of RNA splicing and DNA damage response in 1q21‐amplified multiple myeloma. Cancer Cell. 2017;32:88‐100. 28669490 10.1016/j.ccell.2017.05.011 PMC5593798

[42] Nahi H , Vatsveen TK , Lund J , et al. Proteasome inhibitors and IMiDs can overcome some high‐risk cytogenetics in multiple myeloma but not gain 1q21. Eur J Haematol. 2016;96:46‐54. 25779478 10.1111/ejh.12546

[43] An G , Xu Y , Shi L , et al. Chromosome 1q21 gains confer inferior outcomes in multiple myeloma treated with bortezomib but copy number variation and percentage of plasma cells involved have no additional prognostic value. Haematologica. 2014;99:353‐359. 24213147 10.3324/haematol.2013.088211 PMC3912967

[44] Ballman KV . Biomarker: predictive or prognostic? J Clin Oncol. 2015;33:3968‐3971. 26392104 10.1200/JCO.2015.63.3651

[45] Zhan F , Colla S , Wu X , et al. CKS1B, overexpressed in aggressive disease, regulates multiple myeloma growth and survival through SKP2‐ and p27Kip1‐dependent and ‐independent mechanisms. Blood. 2007;109:4995‐5001. 17303695 10.1182/blood-2006-07-038703 PMC1885527

[46] Fonseca R , Van Wier SA , Chng WJ , et al. Prognostic value of chromosome 1q21 gain by fluorescent in situ hybridization and increase CKS1B expression in myeloma. Leukemia. 2006;20:2034‐2040. 17024118 10.1038/sj.leu.2404403

[47] Huang J , Zhou Y , Thomas GS , et al. NEDD8 inhibition overcomes CKS1B‐induced drug resistance by upregulation of p21 in multiple myeloma. Clin Cancer Res. 2015;21:5532‐5542. 26156395 10.1158/1078-0432.CCR-15-0254 PMC4804624

[48] Muz B , de la Puente P , Azab F , Luderer M , Azab AK . The role of hypoxia and exploitation of the hypoxic environment in hematologic malignancies. Mol Cancer Res. 2014;12:1347‐1354. 25158954 10.1158/1541-7786.MCR-14-0028

[49] Umezu T , Tadokoro H , Azuma K , Yoshizawa S , Ohyashiki K , Ohyashiki JH . Exosomal miR‐135b shed from hypoxic multiple myeloma cells enhances angiogenesis by targeting factor‐inhibiting HIF‐1. Blood. 2014;124:3748‐3757. 25320245 10.1182/blood-2014-05-576116 PMC4263983

[50] Piret JP , Mottet D , Raes M , Michiels C . CoCl_2_, a chemical inducer of hypoxia‐inducible factor‐1, and hypoxia reduce apoptotic cell death in hepatoma cell line HepG2. Ann N Y Acad Sci. 2002;973:443‐447. 12485908 10.1111/j.1749-6632.2002.tb04680.x

[51] Wolff M , Jelkmanna W , Dunstb J , Deppinga R . The aryl hydrocarbon receptor nuclear translocator (ARNT/HIF‐1β) is influenced by hypoxia and hypoxia‐mimetics. Cell Physiol Biochem. 2013;32:849‐858. 24081025 10.1159/000354487

[52] Yao G , Zhang Q , Doeppner TR , et al. LDL suppresses angiogenesis through disruption of the HIF pathway via NF‐κB inhibition which is reversed by the proteasome inhibitor BSc2118. Oncotarget. 2015;6:30251‐30262. 26388611 10.18632/oncotarget.4943 PMC4745795

