# Supplementary material for: ARNT/HIF‐1β links high‐risk 1q21 gain and microenvironmental hypoxia to drug resistance and poor prognosis in multiple myeloma
Source: Cancer Med. 2018 Jun 21;7(8):3899–911. doi: 10.1002/cam4.1596 (PMC6089175; doi:10.1002/cam4.1596)

ARNT/HIF-1β links high-risk 1q21 gain and microenvironmental hypoxia to drug resistance and poor prognosis in multiple myeloma

Supplemental data

Figure legends

Supplementary Figure S1. *ARNT*/HIF-1β is highly expressed in MM and in association with disease progression and adverse cytogenetic abnormalities. (**A**) The GEP datasets of MM patients enrolled in the Total Therapy (TT) trial, as well as of MM cell lines were analyzed to examine *ARNT*/HIF-1β expression in different groups of samples, including normal donors (ND) and patients with monoclonal gammopathy of undetermined significance (MGUS), newly-diagnosed (NDMM) or relapsed/refractory MM (RRMM), as well as human MM cell lines. (**B-D**) Alternatively, *ARNT*/HIF-1β expression was analyzed in subgroups of patients with or without the indicated cytogenetic abnormalities (CAs), including t(4;14) (*FGFR3*), t(4;16) (*MAF*), and del 17p (*TP53*). $P < 0.05$ for patients with vs without each CA. (**E**) Kaplan-Meier analysis was performed to determine correlation between *ARNT* expression and overall survival (OS) in the subset of MM patients who carried 1q21 gain ($P = 0.028$ for low vs high *ARNT* expression).

Supplementary Figure S2. Expression of *ARNT*/HIF-1β is up-regulated in MM carrying 1q21 gain, as well as bortezomib-resistant cells or after transfection with *ARNT*. (**A**) IHC staining for HIF-1β were performed on bone marrow biopsies obtained undergoing routine diagnostic procedures. Representative microscopic images were shown. Squares indicate the area shown in Figure 2A. (**B**) Western blot analysis was performed to monitor expression of HIF-1α and HIF-1β in drug-naive

U266 cells and their bortezomib-resistant counterparts. (**C**) ARP-1 cells were transiently transfected with *ARNT*1.3 (OE) or empty vector (EV), after which expression of HIF-1β was examined by immunofluorescence (red) and counterstained by DAPI (blue).

Supplementary Figure S3. Hypoxia activates both HIF-1 and NF-κB pathway, conferring resistance to bortezomib. (**A**) U266 cells were exposed to 3 mM lactic acid (LA, left) or incubated under hypoxia (1% O_2, right) for 4 - 24 hrs , after which Western blot analysis was performed to monitor expression of HIF-1α, HIF-1β, TRAF2, as well as phosphorylation of p65 (S536). (**B**) H929 and OPM-2 cells carrying 1q21 gain and RPMI8226 cells without 1q21 gain were exposed to 250 μM $CoCl_2$ for 4 hrs, after which expression of HIF-1α, HIF-1β, TRAF2, as well as phosphorylation of IKKα/β (S176/180) were assessed by Western blot analysis. (**C**) RPMI8226 cells were pre-culture under normoxia (21% O_2) or hypoxia (1% O_2) for 4 hrs, followed by treatment with 5 nM bortezomib (Btz) for additional 24 hrs, after which the percentage of cell death (in parallelogram, including Annexin V/7AAD double positive and 7AAD single positive cells) was determined by flow cytometry after stained with Annexin V-FITC and 7AAD. Representative data of at least three independent experiments were shown. (**D**) H929 and RPMI8226 cells were pre-treated with 3 mM lactic acid (LA), followed by either 10 μM parthenolide (PTL; upper panels) or 10 μM IKK2 inhibitor (IKK2i; lower panels) for additional 2 hrs. After treatment, Western blot analysis was performed to monitor the protein levels of HIF-1α, HIF-1β and TRAF2, as well as phosphorylation of p65 (S536). Representative data of at least three independent experiments were shown.

Supplemental Figure S1

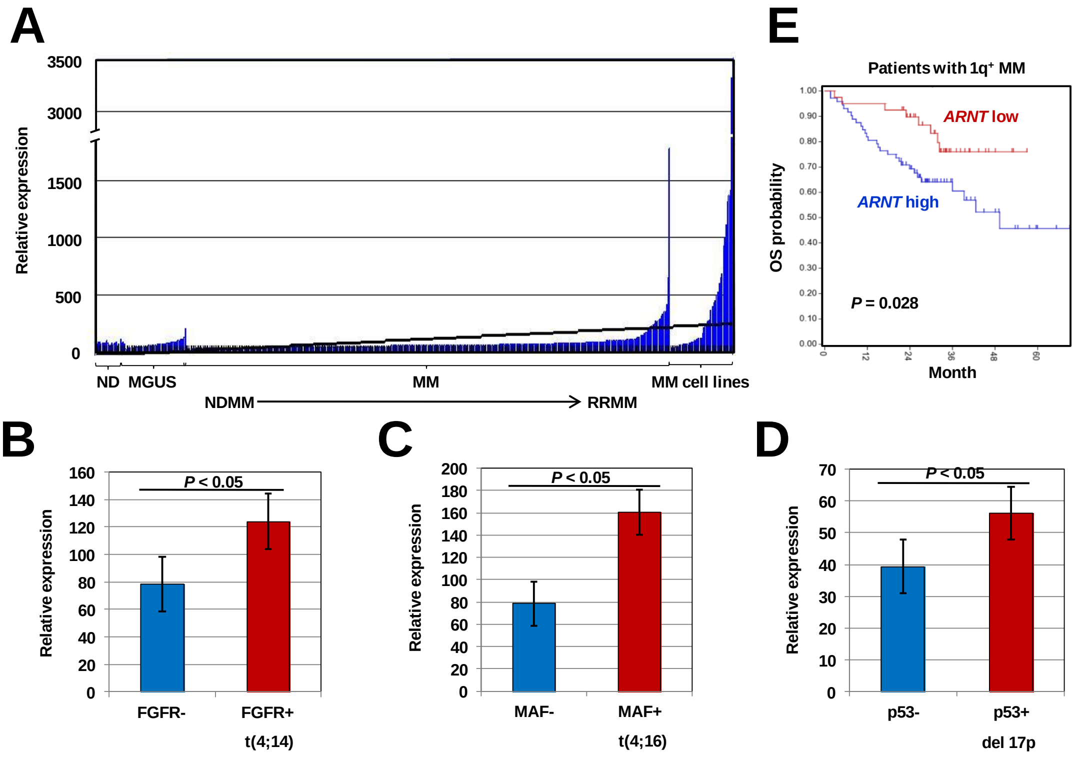

Supplemental Figure S2

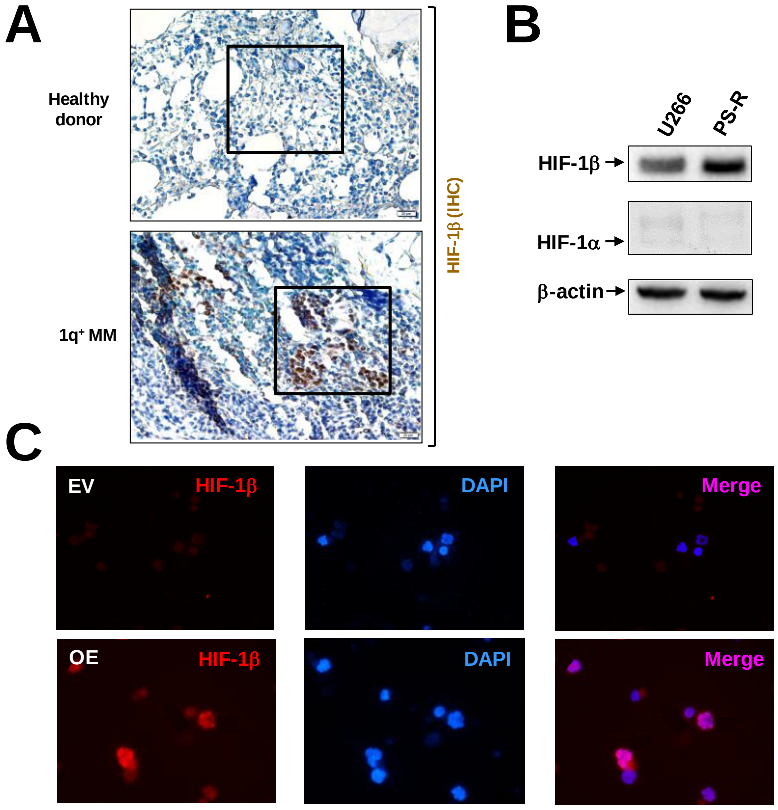

Supplemental Figure S3

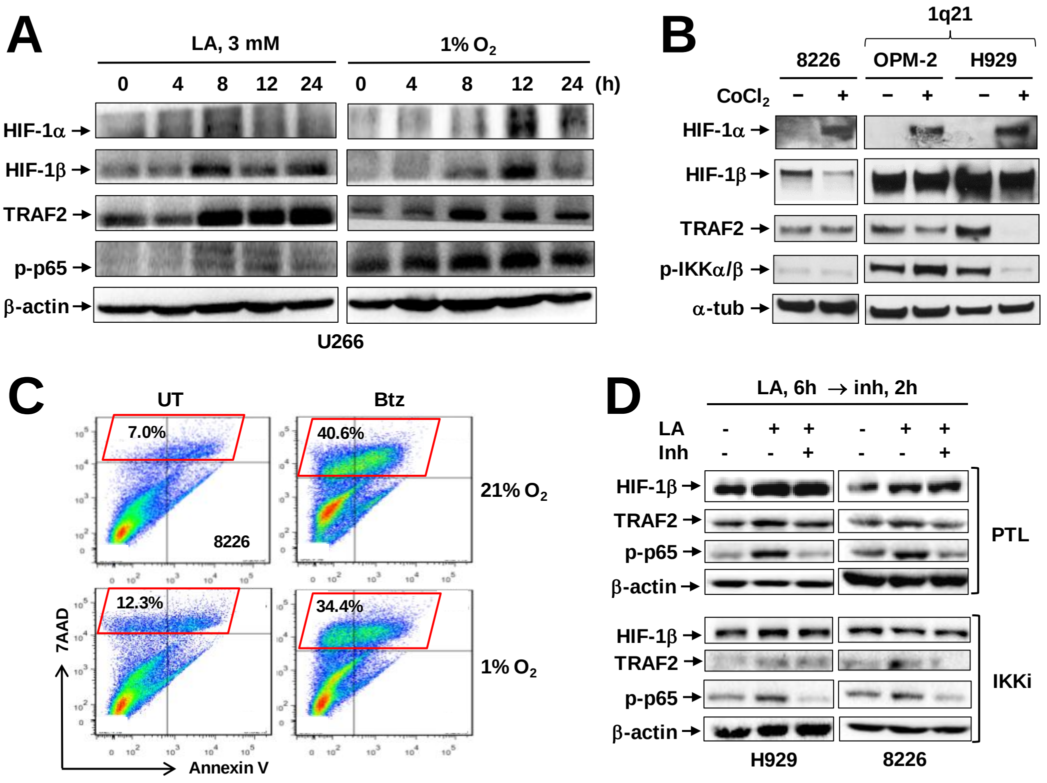

Supplement: Supplementary file 1 [file CAM4-7-3899-s001.pdf]
